# The Transcriptional Landscape and Hub Genes Associated with Physiological Responses to Drought Stress in *Pinus tabuliformis*

**DOI:** 10.3390/ijms22179604

**Published:** 2021-09-04

**Authors:** Tariq Pervaiz, Shuang-Wei Liu, Saleem Uddin, Muhammad Waqas Amjid, Shi-Hui Niu, Harry X. Wu

**Affiliations:** 1 Beijing Advanced Innovation Center for Tree Breeding by Molecular Design, National Engineering Laboratory for Tree Breeding, College of Biological Sciences and Technology, Beijing Forestry University, Beijing 100083, China; tariqzoqi2009@gmail.com (T.P.); liusw2014@126.com (S.-W.L.); saleemkhan86@hotmail.com (S.U.); 2 State Key Laboratory of Crop Genetics and Germplasm Enhancement, Cotton Germplasm Enhancement and Application Engineering Research Center (Ministry of Education), Nanjing Agricultural University, Nanjing 210095, China; waqasamjid@hotmail.com; 3 Umea Plant Science Centre, Department of Forest Genetics and Plant Physiology, Swedish University of Agricultural Sciences, Linnaeus vag 6, SE-901 83 Umea, Sweden; 4 CSIRO National Research Collection Australia, Black Mountain Laboratory, Canberra, ACT 2601, Australia

**Keywords:** *Pinus tabuliformis*, drought stress, drought-recovery, *PtNCED3*, ABA signaling, RNA-Seq

## Abstract

Drought stress has an extensive impact on regulating various physiological, metabolic, and molecular responses. In the present study, the *Pinus tabuliformis* transcriptome was studied to evaluate the drought-responsive genes using RNA- Sequencing approache. The results depicted that photosynthetic rate and H_2_O conductance started to decline under drought but recovered 24 h after re-watering; however, the intercellular CO_2_ concentration (Ci) increased with the onset of drought. We identified 84 drought-responsive transcription factors, 62 protein kinases, 17 transcriptional regulators, and 10 network hub genes. Additionally, we observed the expression patterns of several important gene families, including 2192 genes positively expressed in all 48 samples, and 40 genes were commonly co-expressed in all drought and recovery stages compared with the control samples. The drought-responsive transcriptome was conserved mainly between *P. tabuliformis* and *A. thaliana*, as 70% (6163) genes had a homologous in arabidopsis, out of which 52% homologous (3178 genes corresponding to 2086 genes in Arabidopsis) were also drought response genes in arabidopsis. The collaborative network exhibited 10 core hub genes integrating with ABA-dependent and independent pathways closely conserved with the ABA signaling pathway in the transcription factors module. *PtNCED3* from the ABA family genes had shown significantly different expression patterns under control, mild, prolonged drought, and recovery stages. We found the expression pattern was considerably increased with the prolonged drought condition. *PtNCED3* highly expressed in all drought-tested samples; more interestingly, expression pattern was higher under mild and prolonged drought. *PtNCED3* is reported as one of the important regulating enzymes in ABA synthesis. The continuous accumulation of ABA in leaves increased resistance against drought was due to accumulation of *PtNCED3* under drought stress in the pine needles.

## 1. Introduction

The impact of climate change with the increasing global temperature and long periods of extreme summers have considerably increased the frequent occurrence of drought, resulting tree mortality worldwide [1,2,3]. Forests occupy one-third of the global land, covering more than four billion hectares of the planet and play key role in the water, oxygen, nutrient cycles, carbon sequestration, and various services to protect against environmental degradation [4,5]. The water-conducting mechanism in conifer trees are usually less efficient but safer than those of broad-leaved trees. *P. tabuliformis* is an ecological cornerstone species and important timber species in China and worldwide. The species is mainly distributed in the dry zones of the central and north-western parts of China, and its survival and productivity are heavily dependent on natural rainfall, therefore, strongly affected by various environmental calamities, particularly drought stress [6,7].

Plants cope with drought stress through biochemical, physiological, and molecular responses at whole-plant, cellular, and tissue levels [8,9]. Mechanisms of drought tolerance vary between gymnosperms (needle-leaf) and angiosperms (broadleaf) [6,10]. Coniferous trees have a lower tendency to recover from drought-induced destruction than broad-leaved plants due to the lack parenchymatous and non-structural carbohydrates in xylem tissues [11]. To overcome abiotic stresses, forest plants are dependent on their eternal genetic mechanism, which has been developed over the years of evolution [12,13]. With insufficient water, plants control stomatal conductance to ensure a constant marginal water usage efficiency and avoid carbon gain. The molecular physiology of plants indicates that osmotic adjustment, antioxidative defense, and improved water usage efficiency are an important strategies for enhanced drought tolerance at the cellular and tissue levels [14]. Many metabolic processes, including photosynthesis, are negatively impacted by drought-stress conditions. For example, water deficiency damages the basic structure of the metabolites, which inhibits carbon assimilation and damages photosynthetic activities; and induces many biochemical and physiological responses; therefore, complete physiological recovery is crucial for survival in plants [4,12,15]. Gene expression changes under the drought stress in many model species, such as in arabidopsis, rice, maize, tomato, and soybean [16]. In *Arabidopsis thaliana*, a model plant, physiological and molecular analyses have identified phytohormone signaling as the key to regulating the response against drought or water insufficiency [1]. The drought increases the accumulation of ABA in leaves, resulting in a diminution in stomatal conductance, decreased CO_2_ absorption, and reduced photosynthetic activities. Reactive oxygen species (ROS) are produced due to decreased photosynthesis and impairment of cell components but can also act as an alarm signal that regulates the plant’s defense responses [17]. This excess energy could trigger an increase in ROS production, including O_2_ and H_2_O_2_, which may hinder PSII core subunit, D1, and biosynthesis [18]. The photosynthetic electron transport chain (PETC) activity is also down-regulated in line with the inhibition of D1 synthesis [19]. Several regulatory proteins have been reported to be involved in signal transduction and regulation of stress-responsive gene under drought stress in plants [16,20].

In recent years, the role of the ABA biosynthetic pathway has been extensively investigated due to its synthesis against various stresses. The ABA accumulation begins in the chloroplasts with activation of ABA biosynthesis and hydrolysis of glycosyl ester. In drought stress, the ABA biosynthesis and signaling trigger physiological and biochemical activities [21]. In ABA biosynthetic pathway, the drought-responsive enzymes including 9-cis-epoxycarotenoid dioxygenase (*NCED*), are key enzymes induced under prolonged drought stress [22]. *NCED* is a well-known primary rate-limiting enzyme in the ABA synthesis pathway that catalyzes the first non-reversible step [21]. *PtNCED3* is reported in *Populus trichocarpa* and responds against water dearth. *PtNCED3-2* promoter contains a variety of abiotic stress-responsive elements that function on cis-regulation. In Tobacco ABA content and drought tolerance were limited by RNA-based *NCED3-2* interference silencing. The previous reports also have indicated that the *PtNCED3-2* is concerned with coping with the drought stress and controlling continued plant growth through feedback regulation of the isoprenoid metabolism pathway flux [23].

The present study was designed to understand the gene expression profiles and underlying molecular mechanisms of the ABA singling pathway, accumulation of ABA, and expression of *PtNCED3* under drought stress in *P. tabuliformis* by the RNA-sequencing. The transcriptomic data was used to identify key genes that might be involved in biological processes and elucidate the molecular mechanisms against adverse environmental conditions. We compared the gene expression of abiotic stress-responsive genes under moderate and prolonged drought with the control in *P. tabuliformis.*

## 2. Results

### 2.1. Drought Impact on Photosynthesis and Physiological Indexes in P. tabuliformis

**Figure 1:**
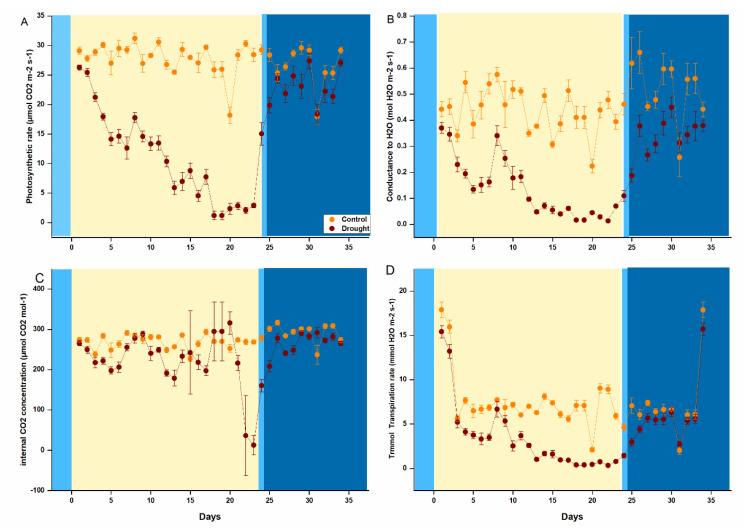
Measurements of Photosynthetic actives of *Pinus tabuliformis* seedlings (**A**) Photosynthetic rate (A_N_), (**B**) Conductance to H_2_O (g**_s_**). (**C**) Internal CO_2_ concentration (C_i_), and (**D**) Transpiration rate (E). All the mentioned parameters were measured by using a portable photosynthesis system (LI-6400T, Li-Cor Inc., Lincoln, NE, USA). Different colors represent irrigation and drought conditions (Blue: Watring, light orange: Drought and Dark Blue: Recovery).

Drought stress affected photosynthesis and physiological parameters including (net photosynthesis (A_N_), stomatal conductance (g_s_), internal CO_2_ concentration (C_i_), and transpiration rate (E). The gas exchange parameters were measured from the first day of drought to the recovery stage of the stressed plants. During this period, A_N_ and g_s_ started to decline as the drought period was prolonged but immediately started to recover after 24 h after re-watering, hence, initiating the recovery of plants. C_i_ was dropped after 20 days of a prolonged drought, but no decline was observed in the control samples (Figure 1). C_i_ concentration was in parallel to control at mild drought stress but decreased significantly under prolonged drought. The transpiration rate (E) consistently decreased under drought compared to control and recovered after re-watering (Figure 1). During the recovery phase, all the physiological parameters were improved, and were parallel to the control (Figure 1).

**Figure 2:**
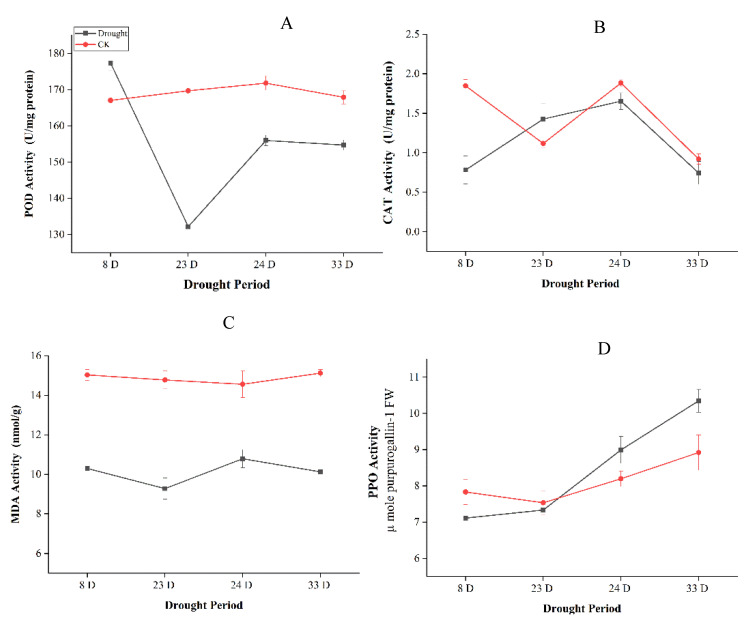
Antioxidant enzyme activities in *Pinus tabuliformis* leaf during drought stress and recovery of (Drought: 8 days, 23 days, Rewatering: 24 days and Recovery: 33 days) (**A**) Peroxidase (POD), (**B**) Catalase (CAT), (**C**) Malondialdehyde (MDA) content, and (**D**) Polyphenol oxidase (PPO).

The activities of antioxidant enzymes peroxidase (POD), malondialdehyde (MDA), catalase (CAT), and polyphenol oxidase (PPO) in leaves were also measured under drought treatment. The total activity of the antioxidant enzymes stayed higher under drought than control samples. POD activity increased initially under drought stress but start decreasing at extreme drought, however, increased after rewatering. MDA levels initially declined due to the severity of the extended drought, but then increased slightly after re-watering, and restored under moderate drought. CAT and PPO were increased with the prolonged drought, although CAT activities were declined after re-watering (Figure 2).

### 2.2. The Global Transcriptomic Response to Drought in P. tabuliformis

To identify essential genes and pathways involved in drought tolerance, we compared the transcriptomic profile of the drought-treated with the well-watered samples in *P. tabuliformis*. The expression of drought-responsive genes changed slightly during the first 8-days under mild drought stress. However, with the increase in the drought severity, the ABA-regulated genes and drought-responsive genes were upregulated. We identified 2192 exhibit differential expression (DEGs) in all 48 samples. Among these DEGs, most showed up-regulation as the drought period prolonged from 8 to 23 days; furthermore, the number of common DEGs were lower in control samples (Supplementary Figure S1). Although after 24 h of re-watering, the expression of drought-responsive genes were decreased (Supplementary data S1). Meanwhile, in the second large cluster, the positively expressed related to metabolic activities in control samples to decline with the onset of drought. However, it started to recover expression level after re-watering to resume normal metabolic activities. Thus, it shows that plants tend to compromise on metabolic activities under drought to survive but resumes metabolic activities in well-watered conditions. It also shows that drought-responsive genes overtake the metabolic genes under drought stress for plants survival.

**Figure 3:**
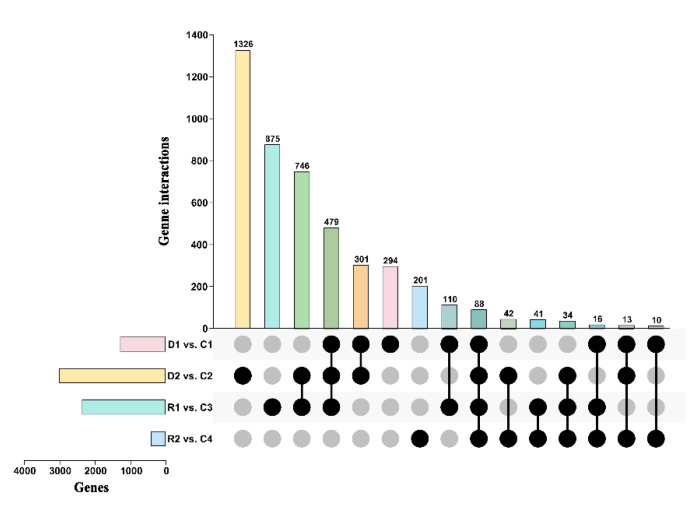
Global gene expression profiles and the identification of DEGs in response to the treatments. The Figure showing shared and unique DEGs of transcriptome associated with drought among drought and recovery stages. The overlap between the differentially expressed genes identified following the control, 8 days of drought, 23 days of drought, and 24 h of re-watering and 10 days of recovery.

The co-expressed 40 genes were recorded among all the comparisons, and it showed that they play a crucial role under stress and normal growth conditions. A significant interaction among treated and control samples was observed; in D2vsC2, 668 were specific genes, followed by R1vsC3 (444), D1vsC1 (158), and R2vsC4 (81). Thus, this shows that the number of specific genes interact with drought stress depending upon severity and length of the drought period for plant survival (Figure 3).

### 2.3. The Drought Responsive Mechanism Is Primarily Conserved between Chinese Pine and Arabidopsis

**Figure 4:**
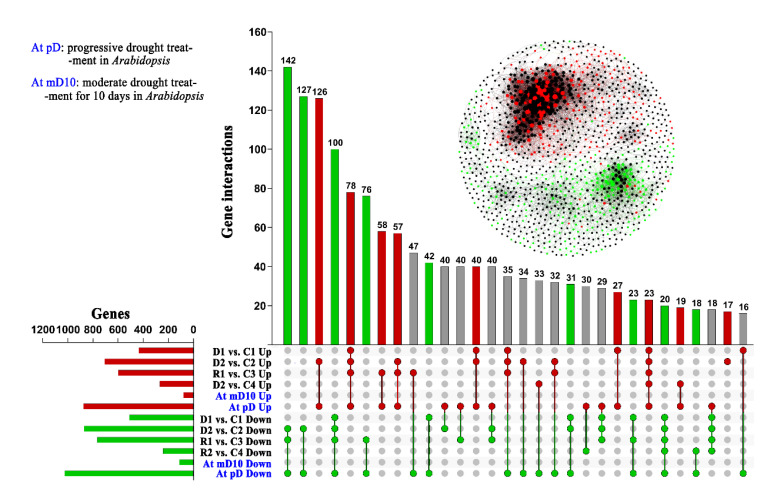
Gene clusters identification in drought-responsive genes and overlap with Arabidopsis: Red and green circles indicate Pt and at conserve drought-response genes, which in the Pt co-expression network, a red dot indicating these gene up-regulated in the D2, and green indicating down-regulated in D2. 70% (6163) genes have a homologous in Arabidopsis, in which 52% homologous (3178 genes corresponding to 2086 genes in Arabidopsis) were also drought-response genes in Arabidopsis.

We compared the transcriptomic profile of Chinese Pine with Arabidopsis through two sides best-hit blast method, at *p* < 0.05 threshold to elucidate drought-responsive conserved mechanism between them. We found 8787 DEGs under drought in Chinese Pine, and found that 70% (6163) of these genes have a homologous in Arabidopsis. Additionally, out of 6163 homologous, 51.57% homologous (3178 genes) in Chinese pine and 33.85% (2086 genes) in Arabidopsis were also drought-responsive (Figure 4). Most of these genes were overlapped under severe drought stress in Chinese pine compared to control and moderate drought stress suggesting their role under severe drought. The current study suggested that the drought-responsive transcripts are mainly conserved between Chinese pine and Arabidopsis, and exhibited similar regulated patterns between *P. tabuliformis* and *Arabidopsis*. D2vsC2 showed the highest up-regulated genes in the compression between drought and control, followed by R1vsC3 and R1vsC1. The number of downregulated genes in D2vsC2 was higher than R1vsC3 and R1vsC1 (Figure 4).

Analysis of promoter motifs showed that ABA signaling pathways could be pretentious with many genes related to drought stress and evolutionary conserved, and might be more interesting to study in *Pinus,* spp. due to its high gene expansion and expression under drought [24]. Previous studies also reported extensive cross-talk related to molecular responses against moderate to severe drought stress [25]. The common homologous genes are highly induced under drought stress in Arabidopsis and pines suggesting these genes are conserved for drought stress responses (Figure 4). The predicted up- and down-regulated genes showed significant changes, in the interaction network; the down-regulated genes were closely clustered compared to the up-regulated ones (Supplementary data S2, *AtDr3178*). Down-regulated genes were highly expressed than up-regulated genes due to being highly conserved and more tightly closed to each other, which shows a strong correlation network among all down-regulated genes. Thus, the results suggested that down-regulated genes might be more highly conserved than up-regulated and non-expressed genes (Figure 4, Supplementary data S2, *AtDr 3178*).

### 2.4. Differential Expression of Transcription Factors, Protein Kinases Families, and Transcriptional Regulators Involved in Drought Stress

**Figure 5:**
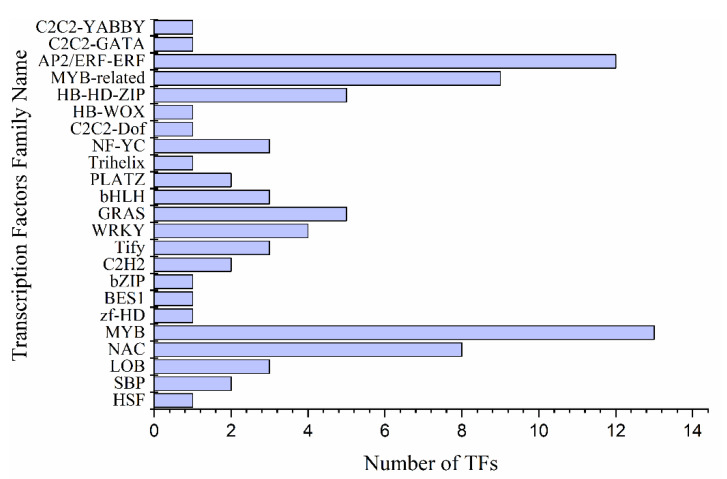
Differentially expressed transcription factors family under drought stress.

A total of 84 transcription factors (TFs) were found in 2192 DEGs under drought stress. A large number of TFs are comprised of 24 families including *MYB* (13) *AP2/ERF-ERF* (12), *MYB*-related (9) and *NAC* (8) were detected. The up-regulated expression of *MYB4* (Pita_unigene7046) was the highest among all recorded *MYB* TFs, followed by *MYB77* (Pita_unigene11287), *MYB3* (Pita_unigene62821) and *MYB79* (Pita_unigene13110) that showed increased accumulation in response to prolonged drought period (Figure 5 and Supplementary data S3). However, the expression of *MYB107* (Pita_unigene22049) was higher in control as compared to drought. Transcriptomic data showed that 51% of *MYB* genes were activated under drought out of which 10% genes were up-regulated and 41% were down-regulated. In Arabidopsis, *MYB* transcription factor genes like *AtMYB2*, *AtMYB74*, and *AtMYB102* showed higher expressions under drought stress [26,27], consistent with the present study. However, *MYB* domain protein 4 (*MYB*-related) (Pita_unigene34156) is highly expressed in the control and during the recovery stage, while, myb-like transcription factor family protein (Pita_unigene62260) expression was increased under drought stress (Figure 5 and Supplementary data S3).

**Figure 6:**
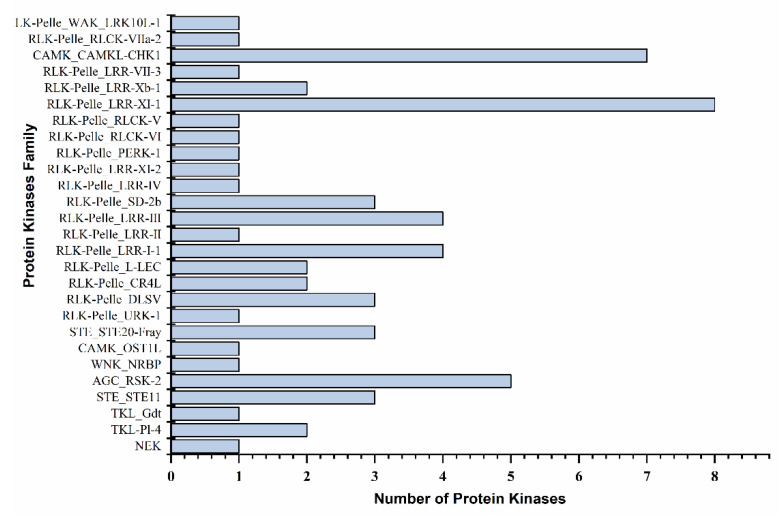
Differentially expressed protein kinases family against drought stress.

**Figure 7:**
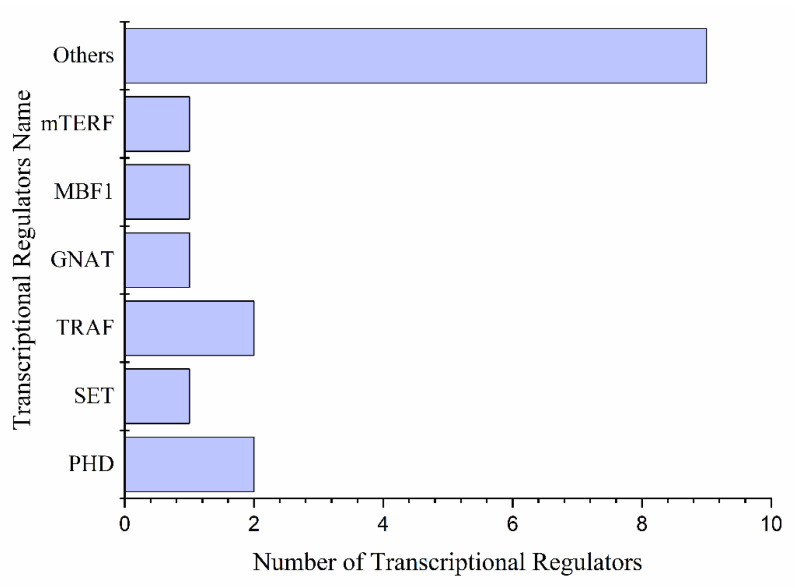
Transcriptional regulators are differentially expressed under drought stress.

Protein kinases (CIPKs) interact with other molecules and play a pivotal function against environmental stresses and adaptation. In the present study, 62 Ppotein kinases were identified (Figure 6 and Supplementary data S4). Most of the protein kinases belonged to 24 families, including leucine-rich receptor-like protein (8), CBL-interacting protein (7), protein kinase superfamily protein (5), leucine-rich repeat protein (4), and leucine-rich repeat transmembrane protein (4) in each. The leucine-rich receptor-like protein expressed 8 PKs, in which *PtTFL2* (Pita_unigene4940 and Pita_unigene49295) showed decreasing expression levels with the severe drought condition. Meanwhile, an increasing trend was observed after 24 h of re-watering and fully recovered plants. The CBL-interacting protein was the second largest number of PKs, in which seven members; including *CIPK15* (Pita_unigene1352) and *CIPK20* (Pita_unigene1353), showed increased expression levels with the drought stress. However, the expression of Ca^2+^ regulated serine-threonine protein kinase (Pita_unigene24122) increased in the recovery stage (Figure 7 and Supplementary data S4). Protein kinase superfamily protein with five members were recorded during the transcriptomic analysis. The *D6PK* family (Pita_unigene59465) was 442.84 folds expression increased under severe drought conditions, with low expression of *PKSF* protein (Pita_unigene17384). Leucine-rich repeat protein expressed with four family members, leucine-rich repeat protein kinase Pita_unigene57577 and Pita_unigene2867 found increasing expression patterns in control and re-watering (Figure 6 and Supplementary data S4).

We found 17 transcriptional regulators involved in drought stress and during the drought recovery of plants. *PHD* (2), *TRAF* (2), *SET* (1), and *GNAT* (1), were found along with nine others (Figure 7). *PHD* *ATXR6* (Pita_unigene39044) showed very low expression levels in all samples under drought and recovery stages, while *AL5* (Pita_unigene8767) showed increased expression levels under prolonged drought and declined after 24 h of re-watering and recovery stage. *SET* (Pita_unigene63770) was highly expressed under mild drought, control, and re-watering stages and showed no expression under severe drought. Expression of *GNAT* with single *TR*, *NAT* (Pita_unigene9031) increased under mild drought and re-watering conditions, but decreased with the severity of the drought. *TRAF* having two members, *BTB/POZ* (Pita_unigene42898) expressed higher in severe drought and gradually decreased with re-watering and recovery. Among others, nine protein kinases with *RR24* (2), *WOL* (2), and *ARR3* (1) almost all had a very low expression under drought (Figure 7 and Supplementary data S5).

### 2.5. Identification of Hub Genes Associated with Control, Drought Stress, and Recovery in Pinus tabuliformis

**Figure 8:**
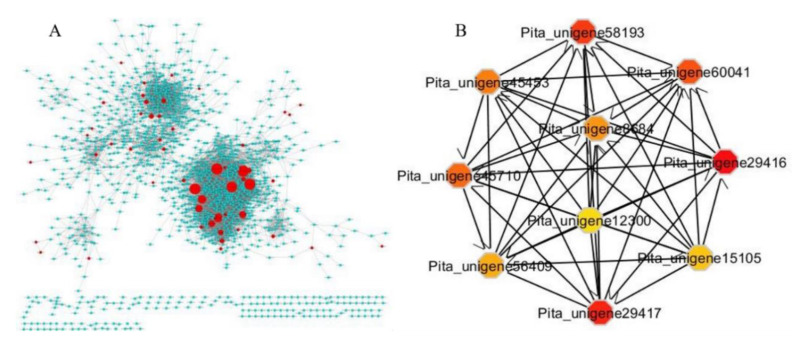
DEGs generate TFs network through Cytoscape ClueGo. (**A**) The networks were constructed based on interactions, co-expression, and gene fusion. (**B**) In every module, the top 10 ranked genes were hub genes, analysis with cytoHubba by degree centrality with a degree score. The size and color change of the nodes denoted the level of degree score.

We identified TFs by Pearson correlation and Cytoscape ClueGo. Further, we screened for collaborative five hub TFs associated with drought stress by constructing a TFCN (transcription factor collaborative network) based gene expression data (TPM, transcript per million) on all of the genes expressed (TPM > 1). In total, 85 TFs were recorded in all samples, and they may have a critical role in the tolerance against drought (Figure 8A).

We found that the co-expressed transcription factors were differentially expressed (*p* < 0.01) in all the samples under drought and recovery stages. The genes expression was predominately the same and closely related to the interaction networks (Figure 8A). Almost all of the TFs in the collaborative network were differentially expressed. CytoHubba was used by degree analysis to predict the hub genes [28]. Finally, Gene Ontology (GO) and Kyoto Encyclopedia of Genes and Genomes pathway (KEGG) were conducted to analyze all of the top 10 differentially expressed hub genes (Figure 8B and Supplementary Figure S2). The key hub genes were chosen with a centrality degree >8. A higher k-core score means a more topological central location. Subnetworks in the TFs were explored by k-core scoring at k-core >6 using the “CytoHubba” package in Cytoscape software. Among five core genes, 2 belong to *GRAM* domain family protein homologous to (Pita_unigene29416 and Pita_unigene29417), and 2 belong to *UDP*-glycosyltransferase 73B4 (Pita_unigene58193 and Pita_unigene45710), and one *NAC* domain transcriptional regulator superfamily protein (Pita_unigene60041). The Pita_unigene8684 is a highly ABA-induced *PP2C* gene (Pita_unigene8684). The *GRAM* domain family protein-containing genes are reportedly linked in membrane-related metabolic processes such as lipid-binding signaling pathways and/or intracellular protein [29,30,31] also reported the response of *GRAM*-domain-containing genes against several abiotic and phytohormone stresses. Furthermore, the *UGT74E2* increased tolerance in arabidopsis against drought and salinity stress and reduced water losses, meanwhile, catalase-deficient plants showed 11 up-regulated *UGTs* in response to water deficit [32,33,34]. Niu, et al. [35] reported that *PtNAC3* belonged to the *NAC* family and was clustered under a subgroup with *ATAF1, ATAF2, ANAC102*, and *ANAC032* in arabidopsis. *ANAC102* was observed to be triggered by various abiotic and biotic stresses [36]. Interestingly, the expression levels of the five TFs in the drought and recovered samples were slightly higher. Most of the hub genes are associated with the ABA signaling pathway. This suggested that the drought tolerance has been improved as the drought cycle is extended and influenced by multiple factors including water and temperatures. The drought impact was reduced gradually upon re-watering and leaves fully recovered after 10 days. Several transcriptomic studies on sorghum using RNA-Seq analysis to monitor gene expression in response to osmotic stress and abscisic acid [37].

### 2.6. ABA Signaling Pathway and *PtNCED3* Expression during Drought Stress

**Figure 9:**
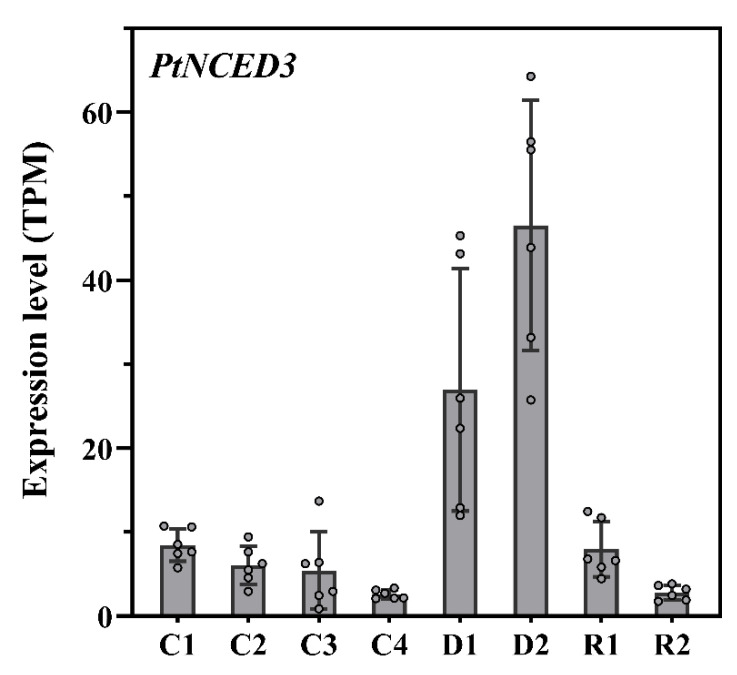
Regulation of transcript abundance related to the ABA biosynthesis pathway. Expression differences of the *PtNCED3* genes between control, under drought, and recovery stages. (C: control, D: Drought, R: Rewatering).

The endogenous abscisic acid (ABA) is a dual modulator regulator against various abiotic stresses for plant growth and responses. ABA signaling pathways regulate the function of many genes under drought stress, but knowledge of interplay among independent and dependent pathways is still limited [38]. Several ABA biosynthesis genes have been cloned, including 9 cis epoxycarotenoids dioxygenases (*NCED*), Zeathanxin epoxidase (ABA1 in Arabidopsis), ABA aldehyde oxidase, and ABA3. ABA is synthesized from b carotene (Tuteja 2007). In the present study, the expression profiles of genes coding key enzymes including, zeaxanthin epoxidase (*ZEP*), ABA aldehyde oxidase (AAO), and ABA-deficient were analyzed, in which two belong to the short-chain dehydrogenase/reductase (*SDR*) family (Supplementary Figure S3). The expression of ABA signaling genes are mainly regulated by the bZIP, with ABRE-binding factors (ABFs) or ABRE-binding proteins (AREB) [38,39]. The RNA-Seq data revealed many ABA-related genes in control, moderate and prolonged drought stress. A total of 27 ABA-related genes were recorded. *PtSnRK2*.*6* (Pita_unigene2665) encodes calcium-independent ABA-activated protein kinase, *PtPP2C1* (Pita_unigene2135) encodes a serine/threonine phosphatase activity *PP2C* protein, which is highly induced by ABA and play crucial roles in the ABA signaling pathway [40,41,42]. They are induced highly by drought. We found that the *PtCYP707A1* (Pita_unigene2481) genes encode a *CYTOCHROME P450* member of the *CYP707A* family with ABA 8′-hydroxylase activity, involved in ABA catabolism, was significantly up-regulated after 24 h of re-watering. Overexpression of *CYP707A1* leads to a decrease in ABA levels in arabidopsis [42,43,44], indicating ABA content is rapidly reduced when drought stress is relieved (Supplementary Figure S4). Among them *PYR1*-like 7 (Pita_unigene21014, Pita_unigene62418, Pita_unigene24111), Polyketide cyclase/dehydrase and lipid transport superfamily protein (Pita_unigene42137, Pita_unigene40730, Pita_unigene5707), *NAC* (Pita_unigene57330, Pita_unigene60041), Transmembrane amino acid transporter family protein (Pita_unigene56161, Pita_unigene22656, Pita_unigene23533), highly ABA-induced *PP2C* (Pita_unigene57214), and *HVA22* homologue E (Pita_unigene8323) were observed with the increasing expression levels. *NCED* is one of the essential proteins due to its role in metabolic activities under abiotic stress and role in ABA biosynthesis. Yang, Zhou, Xu, Li and Zhang [23] reported two variants *NtNCED3-1* and *NtNCED3-2* of the *NCED3* gene in *Nicotiana tabacum*. In the present study, we found that *PtNCED3* from the ABA family had shown different expression patterns under control, mild and prolonged drought and recovery stages. We found the expression pattern was considerably increased with the prolonged drought condition while, *NtNCED3* remained the same under control and re-watered samples (Figure 9). However, the expression was higher in water-deprived samples as the drought period was prolonged. The drought stress samples (D1 and D2) showed highly significant differences from the control (C1-C4), while the recovered samples (R1 and R2) expressed no difference in gene expression. *PtNCED3* showed higher expression under drought stress, while lower expression at the control and recovery stage. The *PtNCED* is a key limiting enzyme due to its role in regulating ABA biosynthetic pathway [19,45]. Although, *OsNCED3* was ectopically expressed in Arabidopsis and contributed to enhanced drought tolerance, increased ABA accumulation, and transformed leaf morphology [46].

### 2.7. Validation of Transcripts by qRT-PCR

To validate the RNA-Seq data, six drought-responsive transcription factors during drought stress were randomly selected to perform qRT-PCR validation (Table 1). Among six, two transcription factors, including Pita_unigene63359 and Pita_unigene39308, showed higher expression under severe drought and the expression increased with prolonged drought until re-watering compared to control and mild drought (Supplementary Figure S5). Furthermore, three transcription factors, Pita_unigene7805, Pita_unigene60666, and Pita_unigene1868 expression was higher under control condition than in moderate and prolonged drought stress. Pita_unigene44994 showed similar expression patterns under control and drought recovery samples (Supplementary Figure S5). The validation results indicated that six selected TFs were induced by drought stress, and hence validated the RNA-seq data.

## 3. Discussion

The present study evaluated the physiological response and molecular regulatory mechanism in *P. tabuliformis* under drought stress. The photosynthetic data (Figure 1) depicted that the accumulation of photosynthates was antagonistic with a moderate and prolonged drought. The photosynthesis and metabolic rate decrease with the decrease in stomatal conductance under mild, prolonged, and severe drought conditions [12]. Because leaf water status declines, when root water uptake is lower than transpirational demand; as a result, the turgor-dependent mechanism activates ABA biosynthesis in leaves [47]. The decrease in photosynthesis further triggers the absorption of excessive light energy, which could cause an increase in the production of reactive oxygen species (ROS), including O_2_ and H_2_O_2_, which impede the biosynthesis of PSII core subunit D1 [18]. Consistently the conductance rate of H_2_O_2_ and the activity of photosynthetic transpiration rate were also downregulated. However, the intercellular CO_2_ concentration was increased under prolonged drought, the interaction between drought and elevated concentration of CO_2_ on Rubisco’s (Vcmax) carboxylation ability and photo-inhibition susceptibility may be a significant determinant of plant responses to seasonal precipitation variations in an anticipated elevated CO_2_ [48]. The intercellular CO_2_ concentration increased under prolonged drought, consistent with the reported facts that an increased CO_2_ concentration would enhance the Rubisco enzyme’s carboxylation and help to increase metabolic activities and inhibition of photorespiration [49].

The antioxidant enzymes POD, SOD, and CAT significantly reduced while PPO activities increased during moderate and severe drought stress in the leaves *P. tabuliformis* (Figure 1). MDA is the first line of protection against the deleterious effects of ROS produced under drought stress, resulting in excessive MDA accumulation [50,51]. MDA accumulation indicates lipid peroxidation of the plant’s strength to tolerate abiotic stresses, including drought and salinity. Under drought stress, plants with higher stress tolerance produce lower MDA content [52].

The ABA regulates plant’s response against abiotic stresses by improving water use efficiency. The ABA accumulation stimulates downstream signaling components and functions by mediating signal cross-talk with other pathways. The ABA pathway is used in many existing schemes to increase drought tolerance [1,53]. ABA-mediated drought stress responses were shown to have a regulatory network of ABA pathway genes and ABA-dependent transcription factors hierarchy [1]. Transcription factors (TFs) have a pivotal role in gene regulation under drought stress. Transcriptomic analysis revealed a total of 84 transcription factors among 2192 DEGs under drought stress. TFs are encompassed in 24 families, including *MYB* (13), *AP2/ERF-ERF* (12), *MYB*-related (9), and *NAC* (8) had the highest number of genes. As in the present study, the higher expression of *MYB* (*AtMYB60* and *AtMYB61*) might be due to involvement in stomatal regulation under drought stress [54,55]. Various TFs families have been identified to be responsible for gene regulation under abiotic stresses; for example, associating with *ABRE* (ABA-responsive cis-regulatory element) in its promoter regions, the essential leucine zipper family (*bZIP*) comprises a common *bZIP* domain for DNA binding at the N terminus and a leucine-rich motif for dimerization at the C terminus and functions in an ABA-dependent manner [56]. Besides, *OsMyb4* integrated with *bZIP* and ABA regulates ABA-responsive genes [57], leading to the upregulation of the drought-responsive genes. The previous reports also indicate that *TaMYB31-B* overexpression in Arabidopsis increased drought tolerance, indicating that the large number of *MYB* TFs increases tolerance under drought stress in pine leaves. *AP2/ERF-ERF* family contains the second-highest number of TFs, suggesting that *AP2/ERF* possibly played a role alongside *BES1* to balance BR-regulated metabolism and drought stress responses. Thousands of BR-responsive genes are regulated by *BES1* and *BZR1* including APETALA2/ETHYLENE RESPONSIVE FACTOR (*AP2/ERF*) [58,59]. We found that *NAC* also has 8 TFs; in response to osmotic stress and drought *NAC* maintained cell turgor pressure, and *ANAC096* cooperated with *ABF2* and *ABF4* [60]. The interaction between *AREB/ABF* and *NAC* under drought strain was observed. *ATAF1*, a *SNAC* TF binding the promoter of *NCED3*, directly regulated the ABA hormone levels and recommended *SNAC’s* role in controlling the expression of ABA-dependent genes [61]. Though the number of *AP2/ERFs* were studied in the abiotic stress-specific regulatory network, the specific capability of *AP2/ERFs* to respond against several stimuli and control multiple stresses enables them to form a more multifaceted stress response network [59]. *AP2/ERF-ERF* family: *ERF8* (Pita_unigene38501) and ERF-1 (Pita_unigene22373) showed high expression levels during severe drought. Furthermore, significant regulation of *NAC* domain-containing protein32 (Pita_unigene60041) and *NAC032* (Pita_unigene59967) was observed in induced drought samples, *NAC* domain proteins are transcription factor family was extensively investigated in crops and are known to regulate metabolic activities in ABA response and against desiccation, drought, and salinity [62,63].

Protein kinases (PKs) play a significant role in plants response to drought and other environmental stresses via activating signal transduction pathways, phosphorylating target proteins, and modifying activity. The protein kinases included RLKs, MAPKs, and calcium/calmodulin-dependent CDPKs. MAPK activity is inhibited and regulated by *PP2C* in plant growth and stress signaling pathways [64,65]. The differential expression of PKs in the study suggests that the PKs are associated with the prolonged drought and regulated in the induced drought conditions (Figure 7). SnRK2 type kinases (SnRK2s) and receptor-like kinases (RLKs) are the most extensively studied protein kinases involved in drought stress tolerance among stress-related kinases. SnRK2s are involved in the ABA central signaling pathway and defense responses such as transcription and stomatal aperture control [65,66]. The PK families RLKs, and FLS2 are reported to trigger the immune response in plants through binding with the pathogen flagellin protein and regulated the stomatal closure induced by ABA through intermolecular interactions with *BAK1*, a co-receptor of flg22 [67,68].

The hub core genes representing *GRAM* domain family protein (AT5G13200, AT5G13200), and *UDP*-glycosyltransferase 73B4 (AT2G15490, AT2G15490) and *NAC* domain transcriptional regulator superfamily protein (AT1G01720) are known to be pivotal regulators in the complex stress-responsive regulatory networks [35,69]. *GRAM* proteins are important for plants responses to abiotic stress, however, their mechanism remains unclear [70]. In this present study, the *GRAM* domain family protein is up-regulated during drought stress. *GRAM* is among the key genes regulating the ABA pathway against abiotic stress (Figure 9). The expression pattern of *GRAM* proteins are mainly associated with the ABA singling, which suggested that *GRAM* might play regulatory functions in the perception of hormone and environmental signaling [30]. *NAC* has pivotal roles in ABA-dependent and independent signaling pathways against drought stress [71]. We found the two *NAC*-related TFs as core genes and expressed differentially under prolonged drought stress. These genes can regulate the ABA signaling pathway and mediate other genes to cope with adverse conditions. The *NCED3* gene expressed in all tested tissues with different response patterns [72] is consistent with our results. In the ABA-independent signaling pathway, *ANAC096* encodes a key *PtNCED3* transcription factor and substantially interacts with the ABA-dependent TFs, i.e., *ABF2* and *ABF4*, to control gene expression against drought stress [60]. Therefore, it is evident from our findings and other reported data that drought tolerance mediated by TFs are more reluctant to ABA signaling pathway during water scarcity and abiotic breakdown.

In the present study, we found ten closely associated core TFs connected with the drought stress levels of ABA (Figure 9). Several ABA biosynthesis genes have been cloned, including 9-cis-epoxycarotenoid dioxygenases (*NCED*), Zeathanxin epoxidase (ABA1 in Arabidopsis), ABA aldehyde oxidase, and ABA3. ABA is synthesized from b-carotene [73]. We found *NAC* as one of the highly regulated genes in mild and prolonged drought compared to control. The expression was increased with the severity of drought; this might be more supportive to tiger the other genes associated with abiotic stress. *NAC* transcription factors regulated stress-responsive genes and were categorized in the abiotic responsive *NAC* (*SNAC*) group. The *NAC* transcription factors can regulate the expression of many genes. *NAC* has a strongly conserved N-terminal DNA-binding domain and variable C-terminal regions, and the C-terminal region is considered to have a critical function in deciding its target genes [74,75]. The present study suggests that *PtNCED3* was strongly expressed under drought stress in all investigated samples. These results are consistent with the previous findings in *P. euphratica* and *P. yunnanensis* in that Unigene21682_All, a putative homolog of *PtNCED3* EST, was up-regulated in response to salt and drought stress respectively [76,77]. Gene expression analysis revealed that seven *SNAC-A* genes were induced by long-term treatment with ABA and/or during age-dependent senescence [75].

## 4. Material and Methods

### 4.1. Plant Material and Drought Treatment

The experiment was conducted at Beijing Forestry University, Beijing China. The *P. tabuliformis* seedlings were planted in 5 cm pots, with the soil composed mixture and organic matter (2:1 *v*/*v*). The climatic conditions were controlled in a glasshouse with the daily average temperature of 28–30 °C (±2) during the drought period (RH 70–80%). We selected 12 *P. tabuliformis* seedlings at the two years and treated them with drought stress under controlled environmental conditions. The controlled seedlings were watered every day up to the field capacity. Physiological parameters were measured for 34 days, and six seedlings were subjected to the drought and control during treatment and control. The irrigation was withheld up to the wilting stage for 23 days, and seedlings were re-watered to recover hydraulic conductivity (Supplementary Figure S6). Simultaneously, six seedlings were kept under control with continuous irrigation up to the field capacity. The leaf samples were collected from each plant for RNA-seq at four time points with four repeats, i.e., 8 days after moderate drought (D1), at the 23 days of drought wilting stage (D2), 24 h (D3) after re-watering, and ten days after recovery (D4), with control at all stages (C1, C2, C3, and C4).

### 4.2. Measurement of Photosynthetic Parameters

An open-flow portable photosynthesis system (LI-6400T, Li-CorInc., Lincoln, NE, USA) with a 6 cm^2^ leaf area chamber with 500 mmol photons m^−2^ s^−1^ of light intensity and with 60% of humidity and humidity, the CO_2_ is stabilized by the CO_2_ supplied to the leaf at 400 ppm or 800 ppm was used to measure the photosynthetic leaf activities during the drought period including, carbon and water gaseous parameters net photosynthesis (AN), stomatal conductance (gs), internal CO_2_ concentration (Ci), and transpiration rate (E).

### 4.3. Antioxidants Extraction

The antioxidants including Peroxidase (POD), and Catalase (CAT), Polyphenol oxidase (PPO), and Malondialdehyde (MDA), were extracted followed by the manufacturers protocol (Nanjing Jincheng Bioengineering Institute, Nanjing, China). The leaf samples of 0.5 g were taken from each treatment and homogenized in 0.05 M phosphate buffer (8 mL, PBS, pH 7.8) with a pre-chilled pestle and mortar. The mixtures were centrifuged at 10,000 RPM for 15 min at 4 °C, and then supernatants were taken were used to determine the malondialdehyde (MDA) content and activities of Polyphenol oxidase (PPO), catalase (CAT), and peroxidase (POD) [52]. Malondialdehyde (MDA A003-3) content was determined by the thiobarbituric acid (TBA) reaction. The mixture was heated at 95 °C in a water bath for 30 min and then quickly cooled in ice followed by centrifugation at 10,000 RPM (10 min), the absorbance of the supernatant at 532 nm was read and the value for the non-specific absorption at 600 nm was subtracted. Catalase (CAT A0071-1) activity was measured as the disappearance of H_2_O_2_ at 240 nm. A 3 mL of reaction mixture contained 2.8 mL potassium phosphate buffer (25 mM, pH 7.0) 0.1 mL H_2_O_2_ (300 mM), and 0.1 mL of enzyme extract. Guaicol peroxidase (POD A084 3-1) activity was determined at 25 °C with guaico (Nanjing Jincheng Bioengineering Institute Nanjing, China). In the presence of H_2_O_2_, POD catalyzes the transformation of guaicol to tetraguaicol. An amount of 2.7 mL potassium phosphate buffer (25 mM, pH 7.0) with 2 mM EDTA, 0.1 mL guaicol (1.5 percent *v*/*v*), 0.1 mL H_2_O_2_ (300 mM), and 0.1 mL enzyme extract is used in the reaction mixture. A spectrophotometer was used to calculate the absorbance at 470 nm for 0.5 min [52].

### 4.4. RNA Library Construction and RNA-Seq Analysis

Total RNA extracted from frozen leaf needles with six replicates of each treatment using Trizol reagent method (Invitrogen, Carlsbad, CA, USA) and subsequently used for mRNA purification and library construction with the Ultra™ RNA Library Prep Kit for Illumina (New England Biolabs, Ipswich, MA, USA) with the prescribed protocol mentioned by the manufacturer [78]. RNA purity was checked using the kaiaoK5500^®^ Spectrophotometer (Kaiao, Beijing, China). RNA integrity and concentration were assessed using the RNA Nano 6000 Assay Kit of the Bioanalyzer 2100 system (Agilent Technologies, Santa Clara, CA, USA). A total amount of 2 μg RNA per sample was used as input material for the RNA sample preparations. Sequencing libraries were generated using NEB Next Ultra™ RNA Library Prep Kit for Illumina (#E7530L, NEB, USA) following the manufacturer’s recommendations and index codes were added to attribute sequences to each sample. Briefly, mRNA was purified from total RNA using poly-T oligo-attached magnetic beads. Using divalent cations under elevated temperature, fragmentation was carried out in NEB Next First Strand Synthesis Reaction Buffer (5_x_).

First-strand cDNA was synthesized using random hexamer primer and RNase H. Second strand cDNA synthesis was performed using buffer, dNTPs, DNA polymerase I, and RNase H. The library fragments were purified with QiaQuick PCR kits and elution with EB buffer, then the terminal repair, A-tailing, and adapter added were implemented. The desired products were retrieved and PCR was performed, and the library was constructed.

### 4.5. Gene Regulatory Network Analysis

The clean reads were mapped to the *P. tabuliformis* reference genome and an abundance of transcripts was estimated using the software application Kallisto [79,80]. Sleuth was used to perfume Differential expression analysis [81], and gene expression patterns were calculated under drought conditions and standardized using Transformation of Z-scores [35,82]. Furthermore, the Cytoscape ClueGo tool was used to analyzed differential expressed genes from the gene network. The network of overlapping and differentially expressed genes was made to amount all samples including mild, prolonged drought and re-watered samples. For each comparison, the Cytoscape add-on ClueGO allows enrichment analysis and the collapse of GO terms into parent categories (Benjamini-Hochberg correction < 0.05). CytoHubba was used by degree analysis to predict the hub genes. Finally, Gene Ontology and Kyoto Encyclopedia of Genes and Genomes pathway analysis analyzed all of the top 10 differentially expressed hub genes. The top 10 high-degree genes were identified by using the CytoHubba plugin [83]. Prediction genes in molecular complex detection of the five top genes ranked in every module were considered core hub genes. The data was obtained for Gene Clusters identification of drought-responsive genes in Arabidopsis were submitted by Harb, et al. [84].

### 4.6. Validation of DEGs

To validate the RNA-Seq data, drought-responsive transcription factors with up- or down-regulation during drought were randomly selected to perform qRT-PCR validation. The seedlings leaf needles from both control and drought were collected and immediately placed in liquid nitrogen and stored at −80 °C. Primers designed for qRT-PCR and Tubulin is used as housekeeping gene are given in Table 1.

## 5. Conclusions

The present study revealed the interactive networks among drought-related transcription factors and the critical hub gens regulating the ABA signaling pathway in pine. We also highlight the role and expression level of *PtNCED3* in mild and severe drought in pine needles. Therefore, the above findings may increase our knowledge of gene regulation under water-deficit and provide a genomic resource to evaluate underlying molecular mechanisms in drought tolerance and develop a strategy to cope with drought stress in pines.

## Figures and Tables

**Table 1:** Differentially expressed genes selected for gene expression analysis by qRT-PCR.

S. No	Gene ID	Gene Function	Primer Sequence
1	Pita_unigene7805	WRKY DNA-binding protein 35 (WRKY35)	GTAGAAACGAGGGAGGGGAG GCTGCCGGAATCTCTCAATG
2	Pita_unigene63359	WRKY DNA-binding protein 57	AGGTCGGTGAACAGAGAAGG CTGCCTGCTGTTCCGATAAC
3	Pita_unigene60666	Encodes WRKY DNA-binding protein 21 (WRKY21).	GGTTGTGTGTGTGCTGTGAT GCTGCAGAATACAAGGAGGC
4	Pita_unigene44994	GRAS family transcription factor	ACAGCTATAGTCTCGTGGGC CCGAAGCTGCTCAAGATCAC
5	Pita_unigene1868	GRAS family transcription factor	ACGGTTCAAGAAAGGACCCA CCACCCAGTTGCAGAGAAAC
6	Pita_unigene39308	MYB domain protein 79	AGTGCCAGTGTCGATCTTGA CCCTTTCAATTGCCTGGCTT
7	Tubulin	Reference gene primer	GGCATACCGGCAGCTCTTC AAGTTGTTGGCGGCGTCTT

## Data Availability

The RNAseq data that support the findings of this study have been deposited in the China National GeneBank Sequence Archive (https://db.cngb.org/cnsa/) of China National GeneBank Database with accession number CNP0002179.

## Supplementary Materials

The following are available online at https://www.mdpi.com/article/10.3390/ijms22179604/s1.
