# Supplementary material for: The Transcriptional Landscape and Hub Genes Associated with Physiological Responses to Drought Stress in Pinus tabuliformis
Source: Int J Mol Sci. 2021 Sep 4;22(17):9604. doi: 10.3390/ijms22179604 (PMC8431770; doi:10.3390/ijms22179604)
Supplement: Supplementary file 1 [file ijms-22-09604-s001.zip › Supplementary Figures.pdf]

Supplementary Figures:

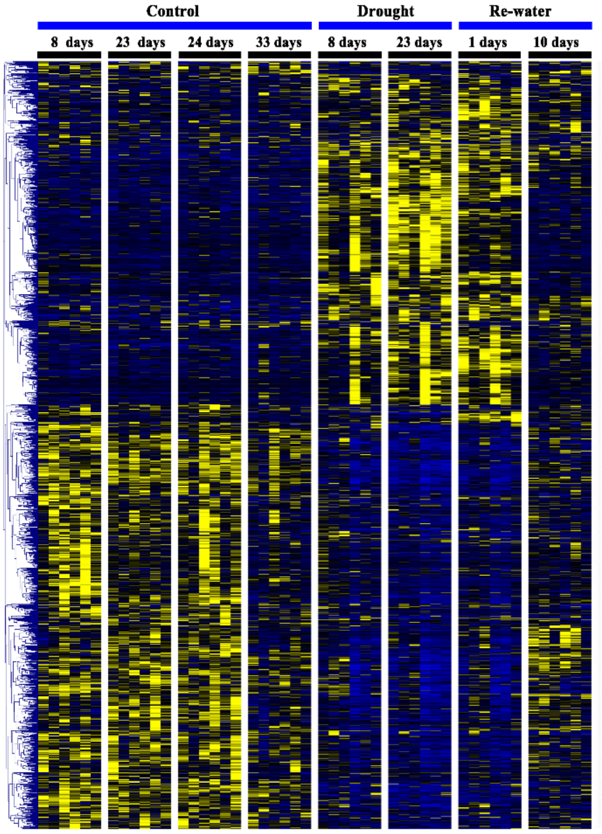

Figure S1. Deferentially expressed genes in response to control, drought and recovery. Heatmap illustrates the Expression profiles of differentially expressed genes between control (Control: 8, 23, 24, and 33 days; Drought: 8 and 23 days; Recovery: 1st and 10 days), Drought (8 and 23 days), and Re-watering (24 and 33 days). Different colors represent different significance levels (Yellow: up-regulated genes and Blue: down-regulated genes).

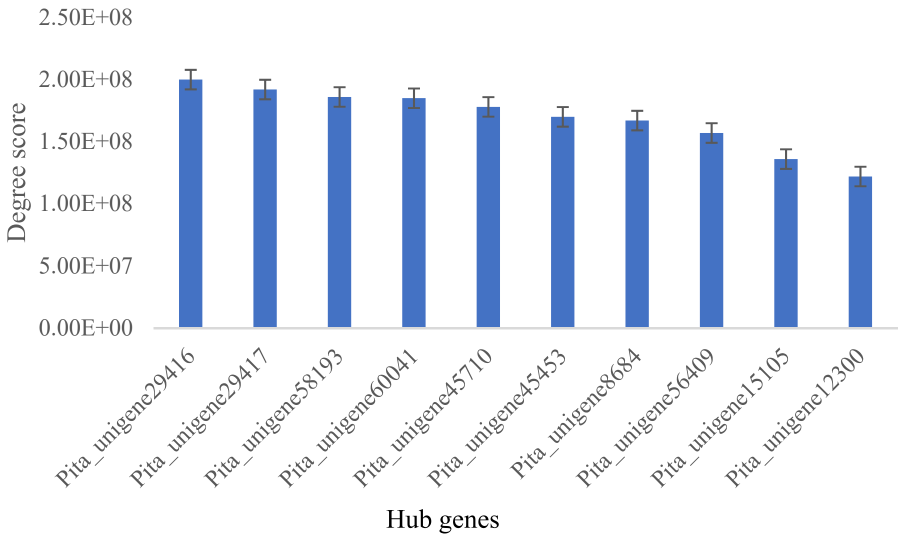

Figure S2: The bar graph shows top 10 ranked genes in every module were considered to be hub genes, analysis with cytoHubba by degree centrality with a degree score.

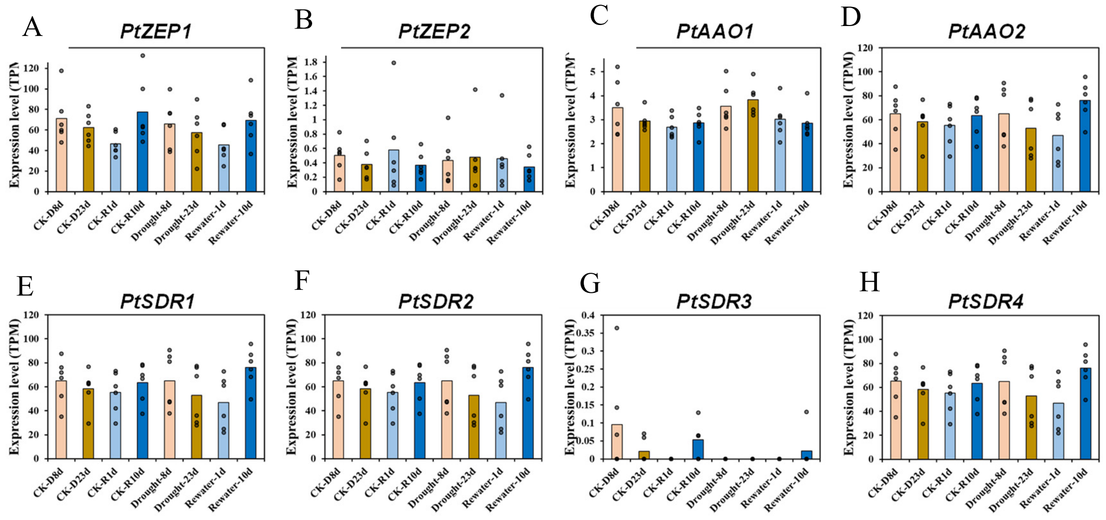

Figure S3: The expression profiles of genes coding key enzymes of the ABA pathway, as zeaxanthin epoxidase (ZEP) (A&B), ABA aldehyde oxidase (AAO) (C&D), ABA-deficient 2 which belongs to short-chain dehydrogenase/reductase (SDR) family (E, F, G, & H).

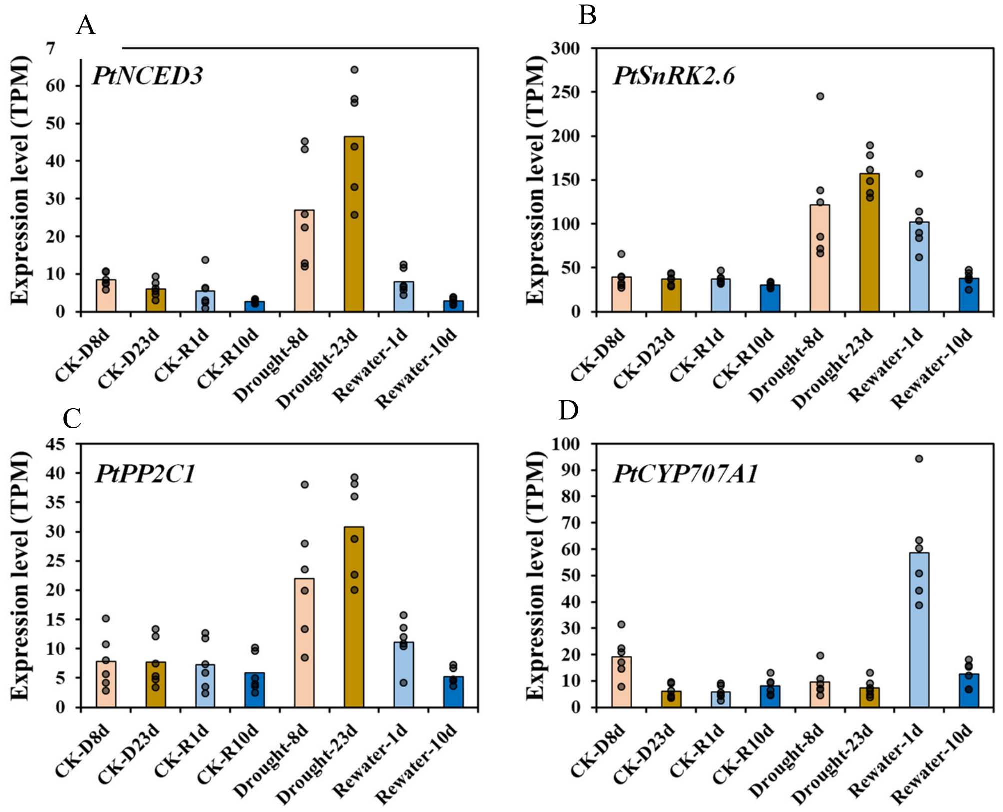

Figure S4: The expression profiles of genes A: PtNCED3, B: PtSnRK2.6, C: PtPP2C1 and D: PtCYP707A1 PtSnRK2.6 encodes calcium-independent ABA-activated protein kinase, PtPP2C1 (Pita_unigene2135) encodes a serine/threonine phosphatase activity PP2C protein, which are highly induced by ABA under drought stress. The CYP707A family with ABA 8'-

hydroxylase activity, involved in ABA catabolism, was significantly upregulated after rewater 1 days.

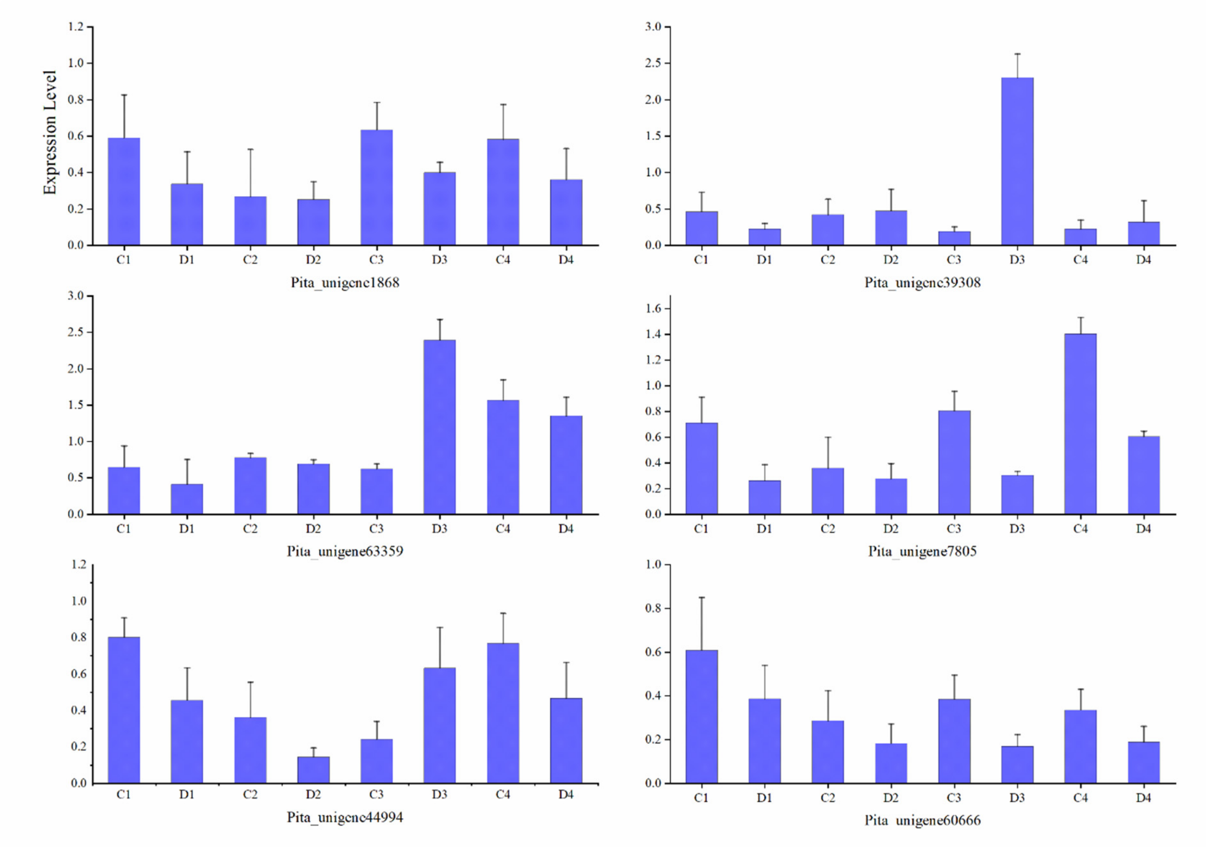

Figure S5: Differentially expressed genes were analysed by qRT-PCR. The expression responses of six transcription factors to control and drought treatment. Four-time points (8 days of drought, 23 days of drought, and 24 hours of re-watering and 10 days of recovery) with six biological replicates were analysed using qRT-PCR. Fold changes [log2(ratio)] of transcript expression levels (Y-axis) in response to drought stress were investigated across a time interval of control, mild drought, severe drought, and rewatering stages (X-axis).

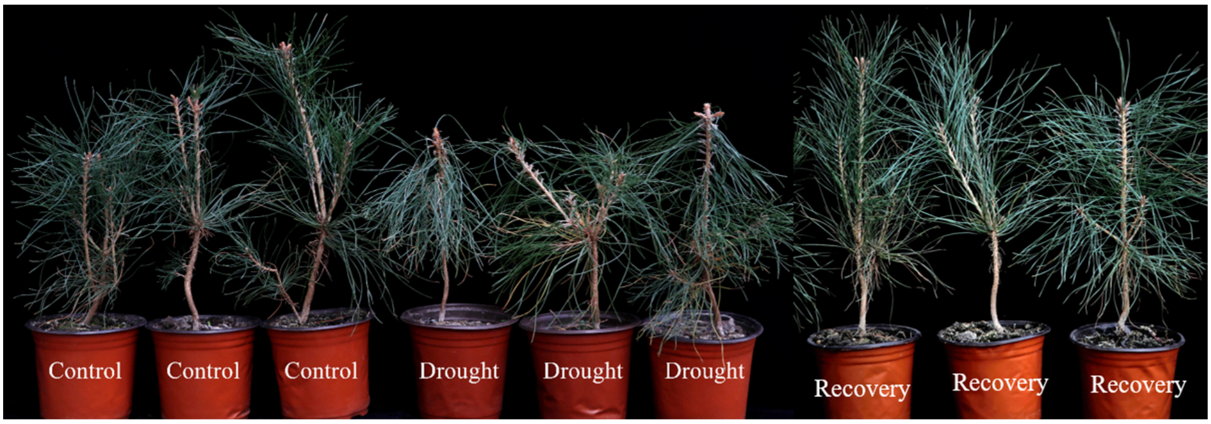

Figure S6: Morphological responses of *Pinus tabuliformis* seedlings under control, drought and recovery stages.
